# Interaction between the estrogen receptor and fibroblast growth factor receptor pathways in non-small cell lung cancer

**DOI:** 10.18632/oncotarget.16030

**Published:** 2017-03-08

**Authors:** Jill M. Siegfried, Mariya Farooqui, Natalie J. Rothenberger, Sanja Dacic, Laura P. Stabile

**Affiliations:** ^1^ Department of Pharmacology and Masonic Cancer Center, University of Minnesota, Minneapolis, MN, USA; ^2^ Department of Pharmacology & Chemical Biology and University of Pittsburgh Cancer Institute, University of Pittsburgh, Pittsburgh, PA, USA; ^3^ Department of Pathology, University of Pittsburgh, Pittsburgh, PA, USA

**Keywords:** NSCLC, FGFR, estrogen

## Abstract

The estrogen receptor (ER) promotes non-small cell lung cancer (NSCLC) proliferation. Since fibroblast growth factors (FGFs) are known regulators of stem cell markers in ER positive breast cancer, we investigated whether a link between the ER, FGFs, and stem cell markers exists in NSCLC. In lung preneoplasias and adenomas of tobacco carcinogen exposed mice, the anti-estrogen fulvestrant and/or the aromatase inhibitor anastrozole blocked FGF2 and FGF9 secretion, and reduced expression of the stem cell markers SOX2 and nanog. Mice administered β-estradiol during carcinogen exposure showed increased FGF2, FGF9, SOX2, and Nanog expression in airway preneoplasias. In normal FGFR1 copy number NSCLC cell lines, multiple FGFR receptors were expressed and secreted several FGFs. β-estradiol caused enhanced FGF2 release, which was blocked by fulvestrant. Upon co-inhibition of ER and FGFRs using fulvestrant and the pan-FGFR inhibitor AZD4547, phosphorylation of FRS2, the FGFR docking protein, was maximally reduced, and enhanced anti-proliferative effects were observed. Combined AZD4547 and fulvestrant enhanced lung tumor xenograft growth inhibition and decreased Ki67 and stem cell marker expression. To verify a link between ERβ, the predominant ER in NSCLC, and FGFR signaling in patient tumors, mRNA analysis was performed comparing high *versus* low ERβ expressing tumors. The top differentially expressed genes in high ERβ tumors involved FGF signaling and human embryonic stem cell pluripotency. These results suggest interaction between the ER and FGFR pathways in NSCLC promotes a stem-like state. Combined FGFR and ER inhibition may increase the efficacy of FGFR inhibitors for NSCLC patients lacking FGFR genetic alterations.

## INTRODUCTION

Lung cancer continues to be the leading cause of cancer related deaths in the United States, with only a 17% five-year survival rate for all stages combined [1]. The evident need for more personalized therapeutics has led to the development of molecularly targeted agents. Specific to the oncogenic pathways and drivers within patient tumor genotypes, targeted therapies were more effective than non-targeted therapies for eligible lung adenocarcinoma cancer patients [2]. However, individual targeted therapies as single agents are clinically useful only in those patients with a known molecular abnormality that constitutes an oncogenic driver. To provide more efficacious therapies for larger patient groups, additional oncogenic pathways and/or selection criteria must be identified for targeted inhibition. Rational combinations of such therapies that can attack interactions among signaling pathways is also a potential strategy for improved clinical utility.

The fibroblast growth factor (FGF) pathway plays an important role in many malignances, including lung cancer [3–8]. The FGF family is composed of 18 ligands and 5 receptors that are involved in essential cellular activities such as proliferation, wound healing, and angiogenesis [3]. Dysregulation of this pathway has been shown to promote oncogenesis [3]. Amplification of the fibroblast growth factor receptor 1 gene (*FGFR1*) in NSCLC occurs in 22% of lung squamous cell carcinomas and 4% lung adenocarcinomas and correlates to poorer overall survival and shorter disease free survival [4–6]. Activating mutations have also recently been identified in the *FGFR2* and *FGFR3* genes in squamous cell lung carcinomas [7]. Co-expression of FGFRs and their corresponding ligands such as FGF2 and FGF9 has been found within NSCLCs indicating an autocrine mechanism for activation of this proliferative signaling pathway [4]. Targeted inhibition of the FGFR pathway has led to the development of a second generation pan-FGFR inhibitor, AZD4547 [8]. AZD4547 has been shown to block activation of FGFR 1, 2 and 3, with lower ability to block FGFR4 and is currently in clinical trials for patients whose tumors contain FGFR mutations, amplifications, and gene rearrangements [9].

The estrogen receptor (ER) pathway is also involved in lung tumorigenesis and proliferation. Preclinical studies have shown the proliferative effect of β-estradiol (E2) on NSCLC cells both *in vitro* and *in vivo* [10, 11]. Evidence for the role of E2 in lung carcinogenesis comes from the Women's Health Initiative, a population study that linked hormone replacement therapy to increased lung cancer mortality [12]. Protection from lung cancer mortality was also observed in breast cancer survivors treated with endocrine therapies [13]. We previously demonstrated that estrogen receptor β-1 (ERβ), the principal ER isoform found in NSCLC, is responsible for mediating proliferative effects of estrogen, while the full length ERα protein is often not expressed [10, 14]. Agents that block estrogen action have been investigated in preclinical models for treatment of lung cancer [15–18], and pathways that show interaction with ER in lung cancer are potential targets for co-targeting.

Recent studies that show interaction between the ER and FGFR pathways in breast cancer [19] suggest co-inhibition of ER and FGFR as a potentially effective therapy. In breast cancer, E2 not only induced increased expression of FGF2, but also enhanced expression of FGF-dependent cancer stem-like cell (CSC) phenotypes [19]. FGFR1 amplification has also been identified as a resistance mechanism to anti-estrogen treatment in certain breast cancers [20]. AZD4547 is currently being evaluated in combination with aromatase inhibitors (AIs) in a clinical trial (NCT01791985) of breast cancer patients who progressed after treatment with AIs as single agents. The hypothesis under investigation in this trial is that combining AZD4547 with other agents will show activity in patients who lack FGFR genetic abnormalities. In the current study, we demonstrate a relationship between the ER and FGFR pathways in NSCLC, using animal models and human cell lines that lack FGFR genetic abnormalities. FGFs and lung stem cell markers were modulated when the ER pathway was either inhibited or stimulated. Co-targeting of the ER and FGFR pathways in NSCLC resulted in greater anti-tumor effects compared to single pathway inhibition, with an accompanying reduction in stem cell markers. The results presented here demonstrate an interaction between the FGF and E2 pathways in lung cancer, and provide support for the hypothesis that clinical utility of a pan-FGFR inhibitor may be improved in NSCLC patients who lack FGFR genetic abnormalities by combination with an agent that blocks the ER pathway.

## RESULTS

### FGFs and stem cell markers in the lungs of mice exposed to the tobacco carcinogen NNK are decreased by treatment with agents that down-regulate the estrogen pathway

**Figure 1:**
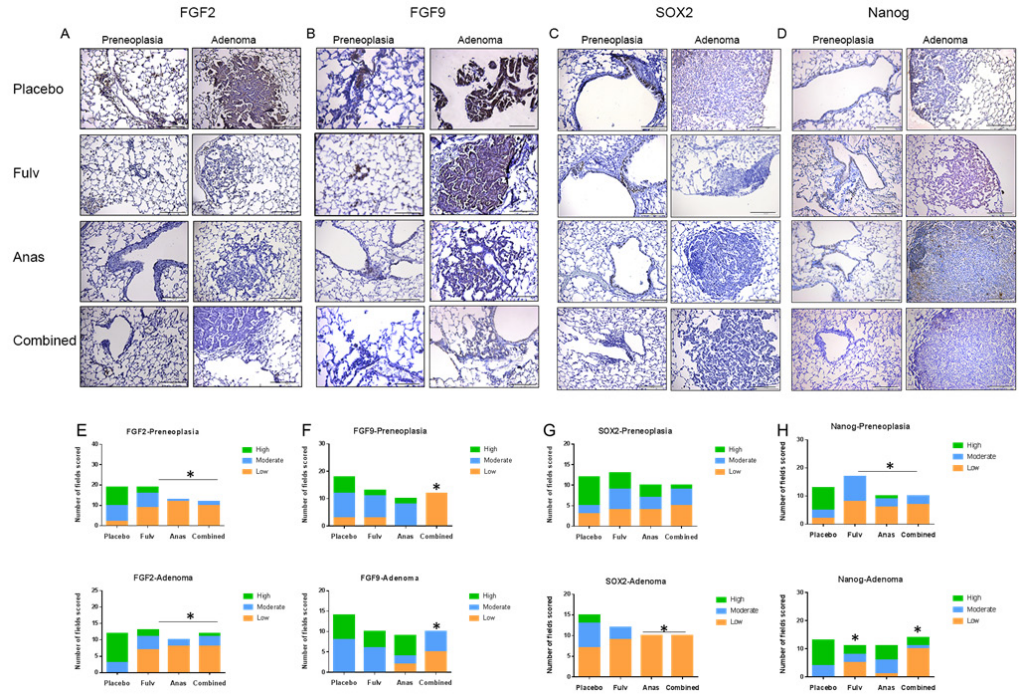
FGF2, FGF9, SOX2, and Nanog expression is decreased in lungs of mice treated with fulvestrant and anastrozole Representative IHC images are shown for FGF2 **A**., FGF9 **B**., SOX2 **C**., and Nanog **D**. from FFPE mouse lungs, which had previously been used for tumor assessment [16]. Preneoplasias and adenomas images were captured at 20X magnification and a representative image is shown for each protein (scale bar = 143μm). (E-H) Multiple images were captured from 4 FFPE blocks for each treatment group. The distribution of number of fields with each score (low, moderate, high) were plotted by treatment group for FGF2 **E**., FGF9 **F**., SOX2 **G**., and Nanog **H**.. A Chi-square test was performed to determine if statistical differences were present across the treatment groups (cutoff *P* < 0.05 or better), followed (if significant) by individual Fisher's exact tests to determine which treatments were significantly different. See Methods for details on scoring assessment. **E**. * *P* < 0.003; **F**. *P < 0.0002; **G**. *P < 0.02; **H**. *P < 0.05 or less (see text).

In our previous published study [16], ovariectomized female mice received treatments with the tobacco carcinogen 4-(methylnitrosamino)-1-(3-pyridyl)-1-butanone (NNK) along with daily androstendione supplementation, the substrate for aromatase, to place estrogen production under control of tissue aromatase. We showed that tissue aromatase is expressed in the lungs, especially in pulmonary macrophages [16]. The AI anastrozole and/or the anti-estrogen fulvestrant were administered as described and the published data showed that these agents, especially in combination, effectively inhibited NNK-induced lung adenoma formation by up to 75%, and also reduced the number of preneoplasias [16]. To determine involvement of the FGF pathway and stem cell phenotypes in these effects, expression of two FGFs thought to be important in lung development, FGF2 [4] and FGF9 [21], and the lung stem cell markers SOX2 and Nanog [22], were examined by immunohistochemistry (IHC) (Figure 1A-1D). Formalin-fixed, paraffin embedded (FFPE) lung tissues that were previously collected after 15 weeks of treatment (placebo; daily 0.1 mg/kg anastrozole; 30 mg/kg fulvestrant given twice per week; or anastrozole plus fulvestrant) [16], were used for IHC determinations. The FFPE lung blocks examined by IHC for FGF2, FGF9, SOX2, and Nanog were from the same animals in which the published reduction in lung tumor formation was observed [16]. Adenomas and preneoplasias were scored separately for each protein, and the scores of multiple microscopic fields from four animals per treatment group were combined (Figure 1E-1H). After treatment with fulvestrant, anastrozole, or the combination, both preneoplasias and adenomas showed reduced staining for FGF2 (Figure 1A), FGF9 (Figure 1B), and Nanog (Figure 1D). For SOX2, staining was decreased in adenomas after fulvestrant, anastrozole, and the combination (Figure 1C), but this effect was not as strong in preneoplasias (Figure 1C). Distribution of IHC scores (low, moderate or high grade staining) displayed significant differences across treatment groups compared to placebo (Figure 1E-1H), with a shift to low and moderate IHC scores after treatment with endocrine agents.

In the case of FGF2, the proportion of fields with a high score in adenomas was reduced from 9/12 (75%) with placebo treatment to 2/13 (15.4%) for fulvestrant alone, 0/10 (0%) for anastrozole alone, and 1/12 (8.3%) with combination treatment, while the proportion of low grade scores increased with treatments (Figure 1E, *P* < 0.003 for all treatment groups). A similar result was observed for preneoplasias (*P* < 0.003 for all groups). For FGF9 (Figure 1F), the combination of anastrozole and fulvestrant showed a significant reduction of high scores in preneoplasias (*P* < 0.0002) and in adenomas (*P* < 0.0002), while single treatments were not significantly different from placebo. Combination treatment (that blocked both the ER and the production of E2) also was significantly different from single treatments (*P* < 0.02).

For Nanog (Figure 1H), all treatments were associated with decreased IHC scores (*P* < 0.05) in preneoplasias, while in adenomas fulvestrant was effective (*P* < 0.024), but not anastrozole (n.s.), and the combination treatment was also effective (*P* < 0.0002). No differences in distribution of SOX2 IHC scores were observed in preneoplasias, while in adenomas anastrozole alone or in combination with fulvestrant reduced the IHC scores (Figure 1G; *P* < 0.02). Similar effects showing reduction in scores for all four proteins after endocrine treatments were observed in the normal bronchial epithelium in the airways in these sections (Supplementary Figure 1). Taken together, these results demonstrate that both FGF ligands and stem cell markers are reduced in murine airways, preneoplasias, and adenomas by agents that block the estrogen pathway, and combination endocrine treatment enhanced some of these effects.

### Exposure to β-estradiol (E2) increases FGFs and stem cell markers in the lungs

**Figure 2:**
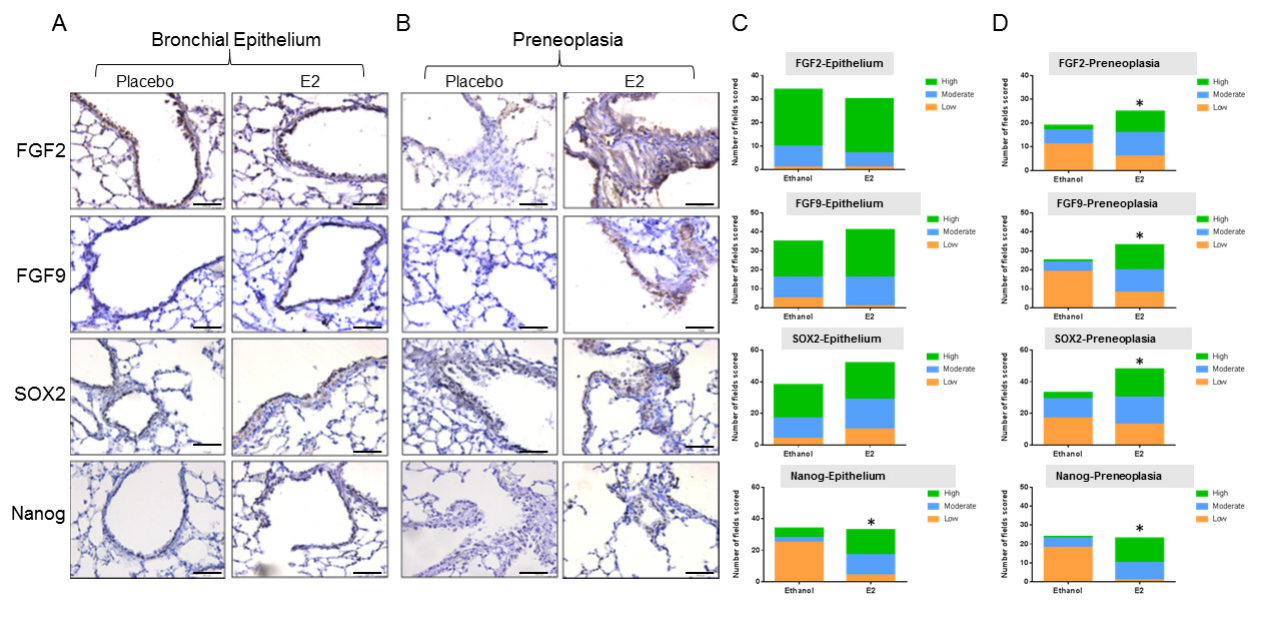
Exposure to E2 in the drinking water increases the expression of FGFs and stem cell markers in the lungs of male mice **A**. Representative IHC staining of bronchial epithelium and **B**. preneoplasias for FGF2, FGF9, SOX2 and Nanog with and without E2 exposure in male mice (scale bar = 71.4μm). **C**. Distribution of scores for low, moderate and high staining, differences assessed by Chi Square test. See Methods for details on scoring assessment. **C**. * *P* < 0.0001; **D**. * *P* < 0.044 or less (see text).

The previous experiment examined the effect of blocking the estrogen pathway in mice exposed to NNK. To determine if activating the estrogen pathway can produce the opposite effect on FGFs and stem cell markers in the lungs, male mice were exposed to NNK and were simultaneously given placebo or E2 in the drinking water for 4 weeks. Male mice, that are known to express ERβ in the airways [23], were used to minimize effects of circulating E2 found in females. Lungs were harvested and IHC was performed for FGF2, FGF9, SOX2, and Nanog in multiple lung sections (Figure 2). In normal airway epithelium (Figure 2A), only Nanog was significantly increased by E2 exposure (*P* < 0.0001, Figure 2C), while FGF2, FGF9 and SOX2 were unchanged. In preneoplasias that developed with NNK treatment, significant differences were observed for all four proteins with E2 exposure. There was increased staining (Figure 2B and 2D), as evidenced by a shift to a higher proportion of grade 2 and/or grade 3 scores for FGF2 (*P* < 0.044), FGF9 (*P* = 0.0002), SOX2 (*P* = 0.02), and Nanog (*P* = 0.0003). E2 treatment also increased the number of NNK-induced lung preneoplasias by 77.5% compared to placebo (50.4% of airways examined (*n* = 226) contained preneoplasias after E2 treatments compared to 28.4% of airways examined (*n* = 227) with placebo, *P* = 0.032) (Supplementary Figure 2). We verified that lungs from these male mice expressed ERβ in the airway epithelium, and this expression was not modulated by E2 exposure (not shown).

### FGFR protein expression and ligand release in NSCLC cell lines

To determine if there is an interaction between the FGF and E2 pathways in human NSCLC, the expression of FGFR family members was determined in five NSCLC cells that lack FGFR1 copy number changes. Protein expression in cell lysates was determined by immunoblotting (Supplementary Figure 3). NSCLC cells without amplification in FGFR1 were chosen because amplification causes constitutive over-activation of the FGFR pathway. Each NSCLC cell line expressed detectable levels of at least three of the four canonical FGFRs, and all were also positive for the decoy receptor FGFRL1 (FGFR5). Cell lines with low FGFR1 protein expressed abundant FGFR2 as well as other FGFRs. All cell lines also expressed ERβ protein (Supplementary Figure 3). Release of FGFs into the growth medium was also measured by ELISA (Supplementary Figure 4) and summarized in Table 1. FGFs 3 and 10 (ligands for FGFR1 and FGFR2) were released at levels from 18.9-30.7 pg/mg protein and 3.3-27.5pg/mg protein, respectively. FGF2 (ligand for all 4 FGFRs) levels ranged from 1.8-49.3 pg/mg protein, while FGF19 (ligand for FGFR4) levels ranged from 9.9- over 1459.6 pg/mg protein (Table 1 and Supplementary Figure 4). FGFs 6 and 9 were minor contributors to the FGF ligands released, and FGFs 4 and 8 were not detected. The presence of a number of different FGFR ligands suggests that all four FGFRs can potentially be activated by autocrine mechanisms in NSCLC, with FGFR1 non-amplified cells secreting ligands that can activate FGFR2, FGFR3 and FGFR4.

**Table 1:** FGF ligand release in NSCLC cell lines

	FGF2	FGF3	FGF4	FGF6	FGF8	FGF9	FGF10	FGF19
273T	++	++	ND	+	ND	+	+	+++
A549	++	++	ND	+	ND	ND	+	++
128-88T	+	++	ND	+	ND	+	+	+
201T	+	++	ND	+	ND	ND	+	++
H23	++	++	ND	+	ND	ND	++	+++

Abbreviation: ND= not detectable; + = 1=10pg/mg protein; ++ = 11-100pg/mg protein; +++= >100pg/mg protein

### β-estradiol induces the release of FGF2 in NSCLC cells

**Figure 3:**
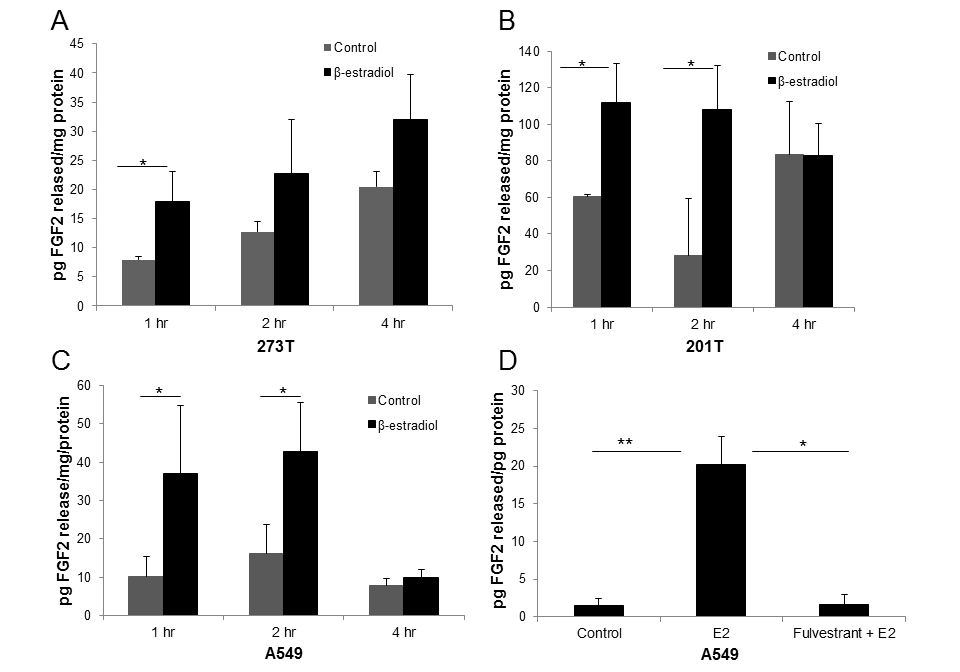
E2 induces FGF2 release in NSCLC cell lines and fulvestrant inhibits E2-induced FGF2 secretion In determining whether E2 treatment results in increased levels of FGF2 ligand release, cells were serum deprived for 48 hr prior to treatment with 10nM E2 or control for 1-4 hr. Media was collected and concentrated. Results were normalized to total protein content. **A**. 273T; **B**. 201T; **C**. A549. **D**. Serum-deprived A549 cells were pre-treated with 5μM fulvestrant for 24 hr followed by E2 treatment for 2 hr. Media and protein lysates were collected and analyzed for FGF2 secretion by ELISA. Results are expressed as the mean ± S.E. pg FGF2/mg protein released. *P < 0.05, **P < 0.01.

FGF2 is known to be an estrogen response gene in breast cancer, and to be released in response to E2 stimulation [18]. To determine whether E2 treatment in NSCLC resulted in increased FGF2 release, FGF2 secretion was measured by ELISA after treatment with 10 nM E2 over 1-4 hr in three cell lines (Figure 3A-3C). A significant increase in FGF2, up to 3.8-fold higher than placebo, was observed in all three cell lines at 1 and 2 hr after E2 exposure. Fulvestrant was also able to block the E2-induced FGF2 release, demonstrating ER-dependence (Figure 3D).

### The anti-estrogen fulvestrant enhances the anti-proliferative effects of FGFR inhibitor AZD4547

**Figure 4:**
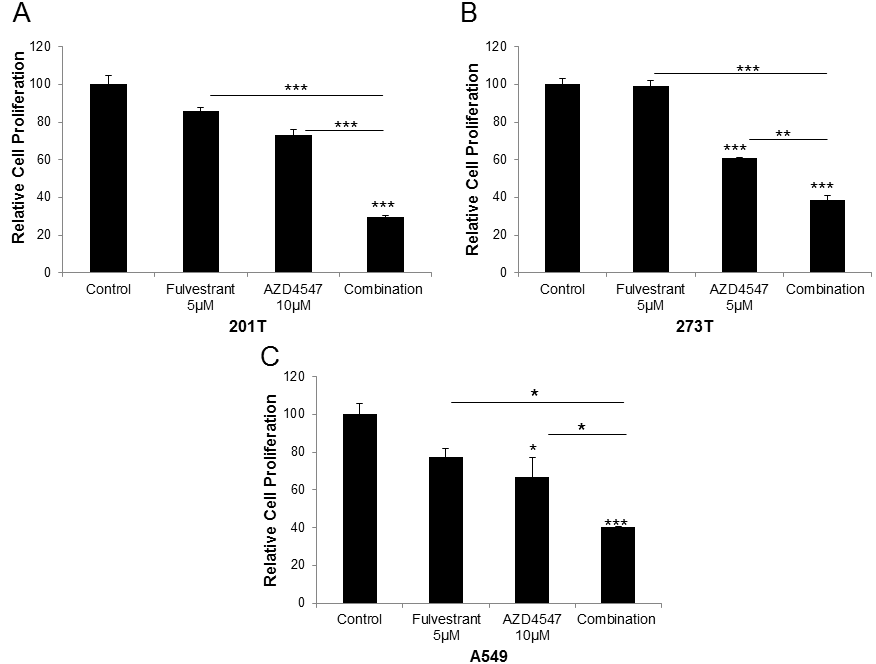
Fulvestrant enhances the anti-proliferative effects of FGFR inhibitor AZD4547 in NSCLC cell lines Cells were treated with 5μM fulvestrant, 5 or 10μM AZD4547 or the combination for 72 hr. Cellular proliferation was measured using the Cell Titer 96 Aqueous One Solution Cell Proliferation Assay. Cell Titer reagent (20μl) was added to each well and plates were incubated for 1 hr. Results are the mean ± S.E. of at least 3 independent experiments each with 6 samples per experimental treatment. ANOVA, * = *P* < .05; ** = *P* < .01;*** = *P* < .001. **A**. 201T; **B**. 273T; **C**. A549.

Based on the hypothesis that the ER and FGFR pathways are interacting in NSCLC, the effect of co-inhibition of both pathways on cellular proliferation was examined. The sensitivity of the cell lines from Figure 3 to the pan-FGFR inhibitor AZD4547 [8] was determined, and a concentration of AZD4547 that yielded a 30-40% inhibition was used alone or in combination with 5 μM fulvestrant (which shows minimal effects as a single agent at this concentration). Enhancement of anti-proliferative effects was observed with combination treatment (Figure 4). In 201T cells, combination treatment produced a 70.7% inhibition compared to control, and 61.4% and 59.9% decreases compared to control were observed in 273T and A549 cells, respectively. The effect of the combination was significantly better than either single treatment in all cell lines (*P* < 0.01 or better, Figure 4).

### Phosphorylation of FRS2 is maximally inhibited by blocking both the ER and FGFRs

**Figure 5:**
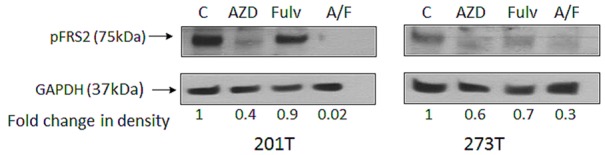
Phosphorylation of FRS2 in presence of fulvestrant and AZD4547 201T and 273T cells were serum starved for 24 hr after plating, followed by addition of DMSO alone (control), AZD4547 (AZD; 2μM), fulvestrant (Fulv; 2μM), or the combination (A/F). Cells were harvested at 72 hr, whole cell lysates were immunoblotted for pFRS2. Expression of GAPDH was used as the loading control and densitometric measurements were corrected for amount of GAPDH.

Since NSCLC cell lines expressed multiple FGFs and FGFRs, we used phosphorylation of the FGFR docking protein fibroblast growth factor receptor substrate 2 (FRS2) [24] as a readout for signaling from the FGFR pathway in the presence of AZD4547 and fulvestrant (Figure 5). Cells were treated with DMSO vehicle, AZD4547, fulvestrant or the combination and cell lysates were probed for phospho-FRS2 (pFRS2). pFRS2 expression was normalized for the housekeeping gene GAPDH and expressed as band intensity relative to control. AZD4547 alone inhibited pFRS2 by 60% in 201T cells and 40% in 273T cells, while fulvestrant showed a lesser ability to decrease phosphorylation (10-30% inhibition). The combination reduced FRS2 phosphorylation by 98% in 201T and by 70% in 273T cells, indicating cooperation between these two pathways to reduce FGFR pathway signaling.

### Combination treatment showed increased anti-tumor effects *in vivo*

**Figure 6:**
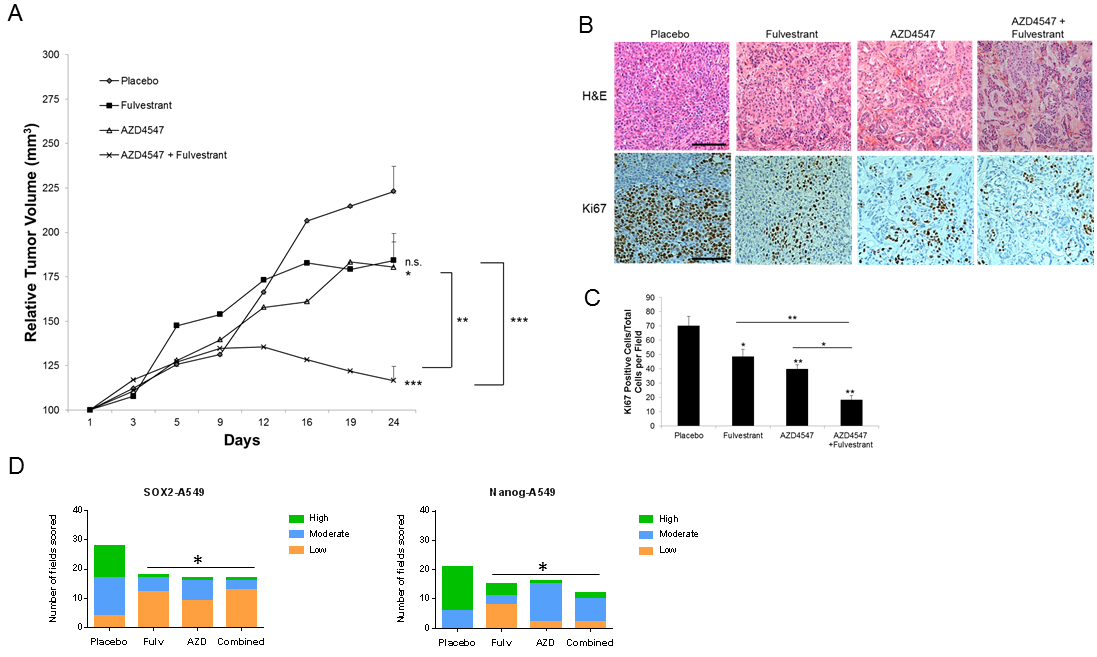
Effect of fulvestrant and AZD4547 on *in vivo* A549 xenograft growth A549 tumor bearing mice received the following treatments for 24 days: placebo, fulvestrant (30mg/kg; s.c.; twice weekly), AZD4547 (12.5 mg/kg p.o.; daily), or combination. **A**. Tumor growth was measured twice weekly and results represent the relative mean tumor volume ± S.E. of 6-8 tumors per treatment group. ANOVA * = *P* < .05; ** = *P* < .01;*** = *P* < .001. **B**. Representative H&E and Ki67 labeling of different treatment groups from xenografts harvested at 24 days (scale bar = 150μm). **C**. Quantitation of Ki67 labeling. Positive cells were counted in 5 high-powered fields per tumor and represent 3 tumors per experimental treatment group. ANOVA ** = *P* < .01;*** = *P* < .001. **D**. Distribution of SOX2 and Nanog IHC scores. SOX2 and Nanog were scored on a scale of low ( < 30% of cells in the field were positive), moderate (30-60% of cells were positive) and high ( > 60% of cells were positive). The distribution of number of fields with each score was plotted in each treatment group. A Chi-square test was performed to determine if statistical differences were present across the treatment groups (*P* < 0.05), followed by individual Fisher's exact tests to determine which treatments were significantly different. SOX2, * *P* < 0.02; Nanog, * *P* < 0.002.

The combination of AZD4547 and fulvestrant was next tested in a xenograft model in mice using ERβ positive cell lines. In A549 xenografts (Figure 6), AZD4547 (daily 12.5 mg/kg), fulvestrant (30 mg/kg twice weekly) or the combination was administered over a 24-day period, beginning when xenografts reached approximately 100mm^3^ volume, after which mice were sacrificed. Tumor volume during the treatment interval was normalized to a 100mm^3^ relative starting volume. After sacrifice, tumors were analyzed using IHC. Fulvestrant and AZD4547 each produced a 33% decrease in tumor volume at 24 days (*P* < 0.05), while the combination yielded an 85% decrease, which was significantly better than either single treatment (*P* < 0.01 or *P* < 0.001, Figure 6A). Tumors also showed a more differentiated histology and a loss of Ki67 labeling after treatment that were maximal with combination treatments (Figure 6B and 6C). In placebo-treated tumors there was a mean of 70 Ki67 positive cells observed per field, while fulvestrant treatment produced a mean of 48.5 positive cells per field, AZD4547 showed 39.8 cells per field, and the combination resulted in only 18.3 positive cells per field (*P* < 0.05 or better compared to single treatments). The stem cell marker SOX2 was also significantly reduced by both fulvestrant and combination treatment compared to placebo (*P* < 0.02), while the shift to lower SOX2 IHC scores with AZD4547 alone was not significantly different than placebo (Figure 6D). For the stem cell marker Nanog, both single and combination treatments significantly reduced IHC scores (*P* < 0.002). Figure 6D shows the IHC score distributions and (Supplementary Figure 5) shows representative tumor sections for SOX2 and Nanog IHC staining.

**Figure 7:**
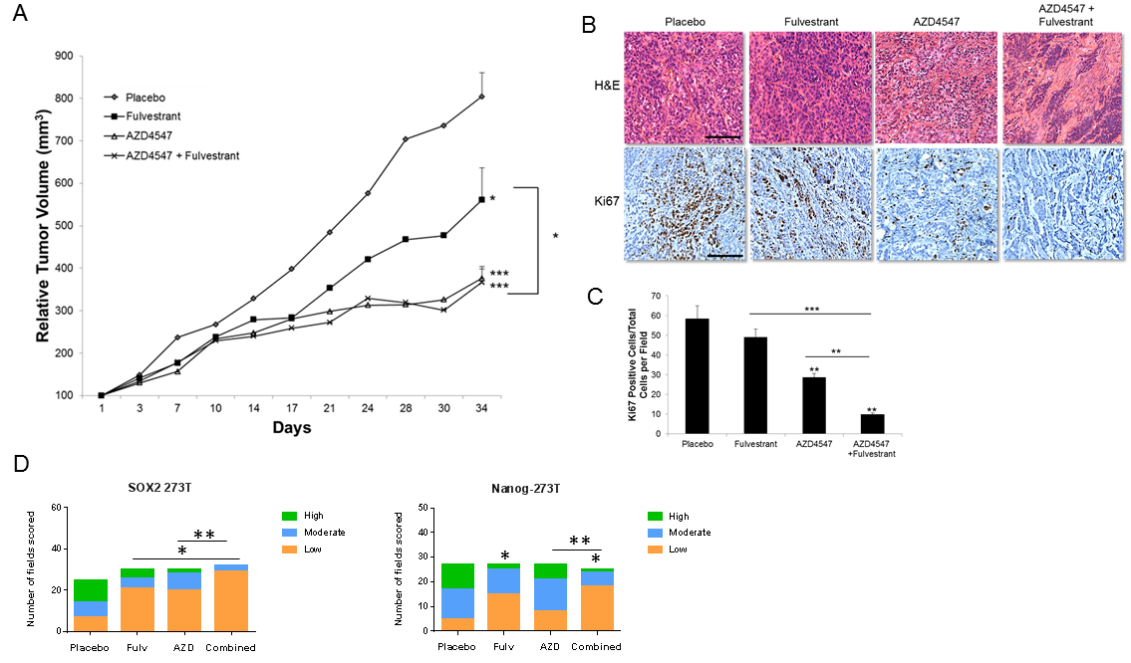
Effect of fulvestrant and AZD4547 on *in vivo* 273T xenograft growth 273T tumor bearing mice received the same treatments as described in Figure 6A for 34 days. **A**. Tumor growth was measured twice weekly and results represent the relative mean tumor volume ± S.E. of 6-8 tumors per treatment group. ANOVA * = *P* < .05; *** = *P* < .001. **B**. Representative H&E and Ki67 labeling of different treatment groups from xenografts harvested at 34 days (scale bar = 150μm). **C**. Quantitation of Ki67 labeling. Positive cells were counted as described in Figure 6C. ANOVA ** = *P* < .01;*** = *P* < .001. **D**. Distribution of SOX2 and Nanog IHC scores. Quantitation and statistical analysis is as described in Figure 6D. SOX2, * *P* < 0.03; ** *P* < 0.05; Nanog, * *P* < 0.004, ** *P* < 0.014.

We repeated this study using 273T xenografts (Figure 7). In this case, fulvestrant inhibited tumor growth based on caliper measurements by 30% (*P* < 0.05), while AZD4547 inhibited tumor volumes by 67% (*P* < 0.001, Figure 7A). The combination gave the same result on tumor size as AZD4547 alone. Examination of the tumor sections after AZD4547 treatment, however, showed a more differentiated tumor histology in which much of the tumor volume was replaced by stroma, with a decrease in malignant cellularity of the tumor of 8% with AZD4547 alone and 25% with the combination of AZD4547 and fulvestrant (Figure 7B). Ki67 labeling confirmed that the combination treatment had a greater effect on S phase labeling of the tumor cells (reduced to a mean of 9.8 cells/field) compared to a mean of 58.3 cells/field with placebo, 49.1 cells per field with fulvestrant, and 28.7 cells per field with AZD4547 (Figure 7B-7C). The effect on Ki67 labeling was significantly greater in combination treatment compared to either single treatment (*P* < 0.01 or better). Staining of 273T xenografts also showed marked reduction in stem cell markers SOX2 and Nanog (Supplementary Figure 5). Individual treatments significantly reduced SOX2 IHC scores (*P* < 0.03, Figure 7D), and the combination of AZD4547 and fulvestrant was very effective, yielding no fields with a high score (0/33), and 88% of fields with a low score (29/33). This combination effect was significantly better than either fulvestrant or AZD4547 alone (*P* < 0.05). For Nanog, fulvestrant also significantly reduced IHC scores (*P* < 0.004), while AZD4547 alone had little effect. The combination treatment yielded IHC scores more distributed toward the low end, with 18/26 (69%) falling in the low scoring group compared to only 4/28 (14%) in the low group with placebo (*P* < 0.004). The distribution of Nanog IHC scores with combination treatment was significantly different than AZD4547 alone (*P* < 0.014). We also evaluated ER expression in the xenografts. While ERα expression was extremely low to undetectable with all treatment groups, ERβ expression was highly expressed with slight reduction with fulvestrant treatment (Supplementary Figure 6).

### The FGFR pathway is altered in patient NSCLC biospecimens expressing high ERβ

To extend these findings to clinical samples, we examined NSCLC biospecimens that varied by ERβ status. We previously published that NSCLC patients with high ERβ protein expression by IHC showed poorer outcome compared to patients with low ERβ expression [25]. To elucidate biological differences between these two groups, an mRNA microarray analysis comparing ERβ low to ERβ high expressing tumors was performed using the Illumina HT-12 V4 Bead Chip (ArrayExpress Accession E-MTAB-3665). mRNA was isolated from lung tumor tissues cut from the same FFPE blocks previously used for the survival study, where sufficient tumor material was available. 165 genes were found to be differentially expressed at *P* < 0.001, and the top 10 differentially expressed genes identified are listed in (Supplementary Table 1). One of the most significantly up-regulated genes in the high ERβ group was *FGFR1* (2.4-fold, *P* = 3.5E-03), while its decoy receptor fibroblast growth factor receptor L1 (*FGFRL1* or *FGFR5*) was one of the most down-regulated genes (0.54-fold, *P* = 8.00E-06). Among the top 10 differentially expressed genes are others that are linked to pulmonary biology and/or lung cancer (*SFTPB*, *SLIT2*, *TNKS*). Pathway analysis demonstrated that these 10 genes form one interacting network (Cancer, Cell Cycle and Organismal Injury, network score of 30), and the top canonical pathways involved in this network are STAT3 signaling (*P* = 0.00054) FGF signaling (*P* = 0.00072), PTEN signaling (*P* = 0.0014), and embryonic stem cell pluripotency (*P* = 0.0018). In this microarray study, mRNA levels of the other FGFRs were not high enough to distinguish expression compared to background, so no comparisons based on ERβ status could be made.

**Figure 8:**
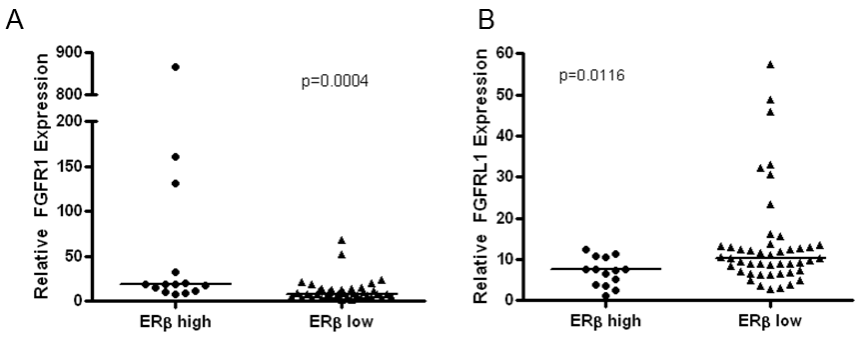
Quantitation of mRNA expression of FGFR1 and FGFRL1 (FGFR5) in NSCLC biospecimens that differ by ERβ status TaqMan qRT-PCR validation of FGFR1 and FGFRL1 expression in the ERβ high/low cohort. Line represents median values in each set and P-values were determined using the Mann-Whitney test.

Quantitative RT-PCR was next done with tumor RNA isolated from the same cohort of biospecimens used for the microarray analysis. As shown in Figure 8, the qRT-PCR findings validated the observed increased levels of *FGFR1* mRNA (*P* = 0.0004) and down-regulation of mRNA for the decoy receptor *FGFRL1/FGFR5* (*P* = 0.0116) in the ERβ high NSCLC tumors compared to ERβ low tumors, confirming the microarray findings of a relationship between the FGFR pathway and ER status in NSCLC.

## DISCUSSION

Targeted therapy has been successful in NSCLC patients when a driver oncogenic alteration has been identified, although many patients eventually develop resistance to these agents. In the case of FGFR inhibitors, they are currently used in patients with an FGFR amplification, gene rearrangement, or mutation. AZD4547, a pan-FGF inhibitor, is currently being evaluated in AI resistant breast cancer (NCT01791985), because of the link between estrogen and the activation of FGF2. These patients lack FGFR genetic alterations, and the hypothesis is that enhanced activity will be seen with the combination of an agent that blocks E2 synthesis and one that blocks the FGFR pathway, even in the absence of an FGFR driver mutation. We sought evidence in this report for such an interaction between the ER and FGF pathways in lung cancer, particularly regarding modulation of markers for a stem-cell phenotype. FGFs are important in lung development, and are needed for branching morphogenesis within the lung and the subsequent development of the airways [21, 26]. Since FGFs, in particular FGF2, are under the control of E2 in breast cancer [19], we examined whether E2 could also be a factor in FGF pathway signaling in NSCLC, which is often positive for ER expression [10, 14, 15, 17, 25].

Our results showed that in lung tissues from female mice in which endocrine agents had a chemopreventive ability against the tumorigenic effect of a tobacco carcinogen [16], FGF2 was reduced by both an ER inhibitor and an AI, while significant reduction in FGF9 required both receptor blockade and inhibition of hormone production. Reduction in FGFs was observed in normal murine airway epithelium, preneoplasias, and lung tumors, suggesting this process is modulated throughout the lung tumorigenesis process. Reduction in FGFs was also accompanied by decreased SOX2 and Nanog stem cell marker expression, which appeared to be less sensitive than individual FGFs to the effect of endocrine therapy, especially in preneoplasias. Single agent treatment with fulvestrant or anastrozole was sometimes ineffective in reducing SOX2 or Nanog in lung tumors, but combination treatment was effective, suggesting that stem cell markers are downstream of changes in FGFs. Stem cell markers were also reduced in the normal airway epithelium by endocrine agents, consistent with a link between FGFs and stem cell markers that is also operative in airways with normal histology.

Exposing male mice to the hormone E2 for four weeks during exposure to a tobacco carcinogen had the opposite effect of endocrine therapies in females. Both FGFs and stem cell markers in the lungs were enhanced by E2 exposure. This effect was observed more markedly in the preneoplasias, which arose in higher numbers in the presence of E2, confirming that E2 had growth-promoting properties in the lung. The normal epithelium was less affected, suggesting some advantage in the development of pre-cancerous changes with overexpression of FGFs and greater expression of a stem cell phenotype. These findings show a relationship between E2 signaling, activation of the FGF pathway in the lungs, expansion of cells with a stem cell phenotype, and promotion of pre-cancerous changes. This observation is in agreement with the reported relationship between E2 and FGFs in breast cancer [19], and with previously reported ability of E2 to promote lung tumorigenesis [11].

In human NSCLC cell lines, we focused on those that expressed ERβ but lacked *FGFR1* gene amplification. We previously observed that NSCLC with amplification of *FGFR1* were not dependent on E2 for release of FGFs and showed no increased anti-tumor effects when FGFR inhibitors were combined with fulvestrant (unpublished observations). This may be because the FGFR pathway is already over-active. In NSCLCs that lack *FGFR1* amplification, we found that multiple FGFRs and FGFs are expressed, even when FGFR1 protein is low, suggesting the pathway is intact, but not dependent on a particular FGFR sub-type. FGF2 release was enhanced by E2, an effect that was blocked by fulvestrant, indicating that ER signaling enhances FGFR signaling in NSCLC. This conclusion was borne out by increased blockage of FRS2 activation and increased anti-tumor effects when an agent that blocks the ER was combined with a pan-FGFR inhibitor. In xenograft studies, the expression of stem cell markers was maximally reduced by the combination of fulvestrant and AZD4547, and this was accompanied by a greater reduction in S phase labeling, showing that the proliferative component of the tumor was reduced when both pathways were interrupted. An increase in the stromal component of the xenografts with combination treatment also suggests that the population of malignant cells was decreased when both the ER and the FGFR were blocked, and replaced by non-malignant cells. Because of the low to negative expression of ERα in the models used, the ER-FGFR interaction in the lung most likely occurs through ERβ.

Results of mRNA profiling comparing NSCLC biospecimens with high and low ERβ protein expression confirmed that FGFR signaling and a stem cell phenotype are positively related to the estrogen pathway. STAT3 and PTEN signaling may be involved in the network of genes that are affected by an ER-FGFR interaction. Taken together, the results of this study suggest that sensitivity of NSCLC to a pan-FGFR inhibitor can be enhanced by combination with an agent that blocks the ER in lung cancers that lack FGFR driver genetic abnormalities. Down-regulating stem cell phenotypes may be an important component of this enhanced effect. Because fulvestrant is relatively non-toxic, it may be an excellent agent to combine clinically with kinase inhibitors where there is evidence of mechanistic interactions between kinase signaling and estrogen signaling.

## MATERIALS AND METHODS

### Cell lines and reagents

NSCLC 201T, 273T and 128-88T cell lines were established in our laboratory as previously described [27]. A549 and H23 cell lines were purchased from American Type Culture Collection (ATCC; Manassas, VA). Cells were cultured in either BME +10% FBS or RPMI 1640 +10% FBS (ATCC; Manassas, VA). Cell lines were authenticated by genotyping with a multiplex STR assay (Genetica) within 3 months of performed studies. E2 and fulvestrant were purchased from Sigma-Aldrich (St. Louis, MO) and diluted in DMSO to 100mM or 5μM stocks, respectively for *in vitro* use. AZD4547 was purchased from ChemieTek (Indianapolis, IN) and diluted in DMSO to a 10mM stock for *in vitro* studies.

### Mouse model of carcinogen-induced lung alterations

FFPE lung tissues were utilized from FVB/N mice treated with the tobacco carcinogen 4-(methylnitrosoamino)-1-(3-pyridyl)-1-butanone (NNK; 24mg exposure over 4 weeks). In Figure 1, we utilized lung tissue previously collected from ovariectomized female mice that were treated with placebo, anastrozole and/or fulvestrant as described previously [16]. These mice were also supplemented with 0.1mg androstendione, the substrate for aromatase, during the entire experiment. For the E2 exposure model in Figure 2, 6 week old male mice were administered NNK (3mg per intraperitoneal injection) twice per week for 4 weeks. During this time, E2 was added to the drinking water at a concentration of 2 μM, solubilized in ethanol. The final concentration of ethanol was 0.001. Controls received drinking water with placebo only (ethanol at 0.001%). The modified drinking water was changed twice per week. Three mice received E2 and three mice received placebo. After 5 weeks, mice were sacrificed and lungs were formalin inflated, paraffin-embedded, and sectioned. 5 micron sections from throughout the lungs, containing all lobes of the lungs, were placed on glass slides and processed for hematoxylin-eosin staining. The number of lung preneoplasias in each treatment group was counted by examining all airways found in 15-18 sections and determining histologically whether each airway showed the presence of hyperplasia. The total number of airways scored was 226 for E2 treatment and 227 for placebo. The scoring was done blinded to treatment group. Animal care was in strict compliance with the institutional guidelines and the protocol was approved by the institutional IACUC committee.

### Protein extraction and western analysis

Basal levels of FGFRs and ERβ were determined by growing cells to 75-90% confluency in T75 flasks. Cells were washed twice with ice-cold PBS and lysed with RIPA buffer (1X PBS, 1% NP40, 0.5% sodium deoxycholate, 0.1% SDS containing 1 protease inhibitor cocktail/10ml buffer (Roche Diagnostics, Indianapolis, IN)). Protein concentration in the supernatant was measured using the BCA-200 Protein Assay Kit (Pierce, Rockford, IL). Equal amounts of protein (25-35 μg) were separated using 7.5% or 10% SDS-PAGE and transferred to PVDF membrane. Membranes were blocked with 10 mM Tris-HCl (pH 7.4), 150 mM NaCl, and 0.1% Tween-20 containing 5% nonfat dry milk at room temperature for 60 min. Primary antibodies included: FGFR1 (1:1000; 3427s; Cell Signaling Technology), FGFR2 (1:500; ab58201; Abcam), FGFR3 (1:1000; ab52246, Abcam), FGFR4 (1:1000; 8562s; Cell Signaling Technology), FGFR5 (1:500; ab95940; Abcam), ERβ (1:500;Clone 68-4; Millipore), pFRS2 (1:1000; ab195826; Abcam), GAPDH (1:5000; 2188s, Cell Signaling Technology) and β-actin (1:5000; Clone C4; Millipore). HRP coupled anti-rabbit or mouse secondary antibodies were applied on membranes for 1-2 hr at room temperature. Protein bands were visualized by chemiluminescence and autoradiography. For the pFRS2 experiment, cells were plated at 75% confluency and serum-starved for 24 hr post attachment. Cells were then treated with DMSO vehicle control or inhibitors as indicated in the legend to Figure 5. Preliminary experiments showed that high baseline pFRS2 declined slowly after treatments, possibly due to autocrine release of FGFs, so inhibition was measured at 72 hr. Cells were harvested and used for immunoblotting as for FGFRs.

### ELISA analysis

Conditioned medium was collected from NSCLC cell lines after 48 hr of incubation at 37°C in serum-free conditions to measure basal secretion of FGF ligands in each cell line. The confluence of each cell line was 90 to 100% at the time of collection. Each sample was concentrated 4-fold using the Amicon^®^ Ultra-4 Centrifugal Filter Devices (Millipore) by centrifuging at 4000 x *g* for 5 minutes at room temperature. The concentrated conditioned media was then analyzed using commercially available ELISA kits for FGF 2, 9, and 19 from R&D systems (Minneapolis, MN), for FGF 3, 6 and 10 from Antibodies Online (Atlanta, GA), for FGF4 from Abcam and for FGF8 from USCN Life Sciences. Protein lysates were also collected for protein concentration normalization. Each assay was performed three times for each sample. For estrogen-induced ligand secretion, cells were serum-deprived for 48 hr prior to 10nM E2 treatment over 1-4 hr. At each time point the media was collected and analyzed as described above. For fulvestrant experiments, fulvestrant was added 24 hr prior to E2 treatment.

### Cell proliferation assay

NSCLC cells were plated on 96 well plates with 10,000 cells per well. The following day, AZD4547 and/or fulvestrant were added at the concentrations indicated in the figures. Cell viability was determined after 3 days of treatment using the Cell Titer Aqueous One Solution MTS assay (Promega, Madison, WI).

### NSCLC cell line xenograft mouse model

Female athymic nude 4-5 week old mice were obtained from Harlan (Indianapolis, IN). A549 or 273T cells were harvested and suspended in a sterile, 50% serum-free PBS/ 50% matrigel (BD Biosciences, San Jose, CA) and cells (1×10^6^) were injected into the rear flank on both sides of each mouse, two injection sites per mouse. When the tumors reached approximately 100mm^3^, mice were divided into four treatment groups: control, AZD45457 (12.5 mg/kg), fulvestrant (30mg/kg) or AZD4547 + fulvestrant. AZD4547 was administered daily by oral gavage at a volume of 0.2ml/ mouse using the vehicle 4% DMSO/30%PEG in deionized water. Fulvestrant or vehicle control (peanut oil) was delivered twice a week by s.c injection. Tumors were measured bi-weekly using digital calipers and reported as a relative tumor volume calculated as (*l* x *w* x *h*) (mm^3^). At the end of the treatment period, the mice were sacrificed and tumors were removed and fixed in 10% formalin buffer for IHC.

### Immunohistochemistry

FFPE mouse lungs and human cell line xenografts were sectioned and mounted on slides. Slides were stained with H&E and evaluated for FGF2, FGF9, SOX2, Nanog and Ki67 expression. After dewaxing in xylene, rehydration and antigen retrieval, endogenous peroxidase activity was blocked with 3% hydrogen peroxide for 15 minutes. Samples were rinsed in PBS followed by blocking with 1.3% goat or mouse serum (MOM kit, Vector Labs). Sections were incubated with primary antibodies overnight in a humidified chamber at 4°C with the following antibodies: FGF2 (MAB5421; 1:200, Abnova, Walnut, CA), FGF9 (ab71395; 1:1000; Abcam), SOX2 (ab97959; 1:1000; Abcam), Nanog (ab80892; 1:400; Abcam) and Ki67 (ab833; 1:50; Abcam). Antibodies recognize both human and murine proteins. HRP labeled secondary antibodies (1:200) were added for 1 hr and developed with DAB-H_2_O_2_ solution (Vectastain Elite HRP stain, Vector Labs). The cell nuclei were stained with Meyer's hematoxylin. For each experimental run, a positive (lung tumor tissue known to be positive for each antigen) and negative control (no primary antibody) was used for each marker. Negative controls were performed by eliminating the primary antibody. Bright field images were captured at 20X or 40X magnification using a 4000B LED microscope and LASv4.7 software (Leica Biosystems). Images were captured from 2-4 sections from each of 4 animals for each treatment groups. Because the incidence of adenoma and preneoplasia varied by treatment, the number of total lesions examined varied from 10-18. FGF2, FGF9, SOX2 and Nanog staining was graded as low ( < 30% positive cells per field), moderate (30-60% positive cells per field) or high ( > 60% positive cells per field), blinded as to treatment group. Examples of low, moderate, and high staining is found in (Supplementary Figure 7). The proportion of each grade for each antigen was analyzed by Chi square analysis using GraphPad PRISM (Version 7). For antigens that showed significance by Chi square test, individual Fisher's exact tests were performed to determine significant differences for each condition compared to placebo, and for combination treatments compared to single treatments. Ki67 cell proliferation index was quantitated as the number of Ki67 positive cells per field divided by the total number of tumor cells per field.

### Microarray gene expression analysis

RNA was isolated using the High Pure FFPE RNA Isolation Kit (Roche) from FFPE sections from lung cancer cases that we have previously identified to have high ERβ (*n* = 15) and low ERβ (*n* = 49) protein expression by IHC [15]. The Illumina Whole-Genome DASL Assay (HT-12 V4 Bead Chip platform) was used to compare mRNA profiles between the ERβ high *versus* low expressing lung tumors. Data was analyzed using BeadStudio software and Biometric Research Branch (BRB) array tools Version 4.2.1 for subsequent analyses. The microarray data is available through ArrayExpress (accession number E-MTAB-3665).Validation of FGFR1 and FGFRL1 mRNA expression in the ERβ high/low cohort was performed using TaqMan q-RT-PCR and primer/probe sets from Invitrogen.

### Statistical analysis

All data are expressed as mean ± S.E. Statistical analyses were performed with GraphPad Prism version 4.03 for Windows (GraphPad Software, San Diego California USA). The statistical significance was determined by unpaired *t*-test, ANOVA, Chi-squared test, or Fisher's exact test with a 95% confidence level (*P* < 0.05).

## SUPPLEMENTARY MATERIALS FIGURES AND TABLES



## Abbreviations

ER: estrogen receptor
NSCLC: non-small cell lung cancer
FGF: fibroblast growth factor
FGFR: fibroblast growth factor receptor
E2: β-estradiol
CSC: cancer stem cell
FFPE: formalin-fixed, paraffin embedded

## References

[1] Society AC (2014). Cancer Facts & Figures 2014. In: Society AC, ed.American Cancer Society.

[2] Kris MG, Johnson BE, Berry LD, Kwiatkowski DJ, Iafrate AJ, Wistuba II, Varella-Garcia M, Franklin WA, Aronson SL, Su PF, Shyr Y, Camidge DR, Sequist LV (2014). Using multiplexed assays of oncogenic drivers in lung cancers to select targeted drugs. Jama.

[3] Brooks AN, Kilgour E, Smith PD (2012). Molecular pathways: fibroblast growth factor signaling: a new therapeutic opportunity in cancer. Clin Cancer Res.

[4] Marek L, Ware KE, Fritzsche A, Hercule P, Helton WR, Smith JE, McDermott LA, Coldren CD, Nemenoff RA, Merrick DT, Helfrich BA, Bunn PA, Heasley LE (2009). Fibroblast growth factor (FGF) and FGF receptor-mediated autocrine signaling in non-small-cell lung cancer cells. Mol Pharmacol.

[5] Seo AN, Jin Y, Lee HJ, Sun PL, Kim H, Jheon S, Kim K, Lee CT, Chung JH (2014). FGFR1 amplification is associated with poor prognosis and smoking in non-small-cell lung cancer. Virchows Arch.

[6] Weiss J, Sos ML, Seidel D, Peifer M, Zander T, Heuckmann JM, Ullrich RT, Menon R, Maier S, Soltermann A, Moch H, Wagener P, Fischer F (2010). Frequent and focal FGFR1 amplification associates with therapeutically tractable FGFR1 dependency in squamous cell lung cancer. Sci Transl Med.

[7] Liao RG, Jung J, Tchaicha J, Wilkerson MD, Sivachenko A, Beauchamp EM, Liu Q, Pugh TJ, Pedamallu CS, Hayes DN, Gray NS, Getz G, Wong KK (2013). Inhibitor-sensitive FGFR2 and FGFR3 mutations in lung squamous cell carcinoma. Cancer Res.

[8] Gavine PR, Mooney L, Kilgour E, Thomas AP, Al-Kadhimi K, Beck S, Rooney C, Coleman T, Baker D, Mellor MJ, Brooks AN, Klinowska T (2012). AZD4547: an orally bioavailable, potent, and selective inhibitor of the fibroblast growth factor receptor tyrosine kinase family. Cancer Res.

[9] Smyth K TN, Pearson A, Peckitt C, Chau I, Watkins DJ, Starling N, Rao S, Gillbanks A, Kilgour E, Sumpter KA, Smith NR, Cutts R (2016). Phase II study of AZD4547 in FGFR amplified tumors: gastroesophageal (GC) cohort pharmacodynamic and biomarker results. J Clin Oncol.

[10] Stabile LP, Davis AL, Gubish CT, Hopkins TM, Luketich JD, Christie N, Finkelstein S, Siegfried JM (2002). Human non-small cell lung tumors and cells derived from normal lung express both estrogen receptor alpha and beta and show biological responses to estrogen. Cancer Res.

[11] Hammoud Z, Tan B, Badve S, Bigsby RM (2008). Estrogen promotes tumor progression in a genetically defined mouse model of lung adenocarcinoma. Endocr Relat Cancer.

[12] Chlebowski RT, Schwartz AG, Wakelee H, Anderson GL, Stefanick ML, Manson JE, Rodabough RJ, Chien JW, Wactawski-Wende J, Gass M, Kotchen JM, Johnson KC, O’Sullivan MJ (2009). Oestrogen plus progestin and lung cancer in postmenopausal women (Women’s Health Initiative trial): a post-hoc analysis of a randomised controlled trial. Lancet.

[13] Bouchardy C, Benhamou S, Schaffar R, Verkooijen HM, Fioretta G, Schubert H, Vinh-Hung V, Soria JC, Vlastos G, Rapiti E (2011). Lung cancer mortality risk among breast cancer patients treated with anti-estrogens. Cancer.

[14] Hershberger PA, Stabile LP, Kanterewicz B, Rothstein ME, Gubish CT, Land S, Shuai Y, Siegfried JM, Nichols M (2009). Estrogen receptor beta (ERbeta) subtype-specific ligands increase transcription, p44/p42 mitogen activated protein kinase (MAPK) activation and growth in human non-small cell lung cancer cells. J Steroid Biochem Mol Biol.

[15] Stabile LP, Lyker JS, Gubish CT, Zhang W, Grandis JR, Siegfried JM (2005). Combined targeting of the estrogen receptor and the epidermal growth factor receptor in non-small cell lung cancer shows enhanced antiproliferative effects. Cancer Res.

[16] Stabile LP, Rothstein ME, Cunningham DE, Land SR, Dacic S, Keohavong P, Siegfried JM (2012). Prevention of tobacco carcinogen-induced lung cancer in female mice using antiestrogens. Carcinogenesis.

[17] Shen L, Li Z, Shen S, Niu X, Yu Y, Li Z, Liao M, Chen Z, Lu S (2012). The synergistic effect of EGFR tyrosine kinase inhibitor gefitinib in combination with aromatase inhibitor anastrozole in non-small cell lung cancer cell lines. Lung Cancer.

[18] Siegfried JM, Gubish CT, Rothstein ME, Henry C, Stabile LP (2012). Combining the multitargeted tyrosine kinase inhibitor vandetanib with the antiestrogen fulvestrant enhances its antitumor effect in non-small cell lung cancer. J Thorac Oncol.

[19] Fillmore CM, Gupta PB, Rudnick JA, Caballero S, Keller PJ, Lander ES, Kuperwasser C (2010). Estrogen expands breast cancer stem-like cells through paracrine FGF/Tbx3 signaling. Proc Natl Acad Sci U S A.

[20] Balko JM, Mayer IA, Sanders ME, Miller TW, Kuba MG, Meszoely IM, Wagle N, Garraway LA, Arteaga CL (2012). Discordant cellular response to presurgical letrozole in bilateral synchronous ER+ breast cancers with a KRAS mutation or FGFR1 gene amplification. Mol Cancer Ther.

[21] Yin Y, Wang F, Ornitz DM (2011). Mesothelial- and epithelial-derived FGF9 have distinct functions in the regulation of lung development. Development.

[22] Park E, Park SY, Sun PL, Jin Y, Kim JE, Jheon S, Kim K, Lee CT, Kim H, Chung JH (2016). Prognostic significance of stem cell-related marker expression and its correlation with histologic subtypes in lung adenocarcinoma. Oncotarget.

[23] Beyer C, Kuppers E, Karolczak M, Trotter A (2003). Ontogenetic expression of estrogen and progesterone receptors in the mouse lung. Biol Neonate.

[24] Delpuech O, Rooney C, Mooney L, Baker D, Shaw R, Dymond M, Wang D, Zhang P, Cross S, Veldman-Jones M, Wilson J, Davies BR, Dry JR (2016). Identification of Pharmacodynamic Transcript Biomarkers in Response to FGFR Inhibition by AZD4547. Mol Cancer Ther.

[25] Stabile LP, Dacic S, Land SR, Lenzner DE, Dhir R, Acquafondata M, Landreneau RJ, Grandis JR, Siegfried JM (2011). Combined analysis of estrogen receptor beta-1 and progesterone receptor expression identifies lung cancer patients with poor outcome. Clin Cancer Res.

[26] Warburton D, Perin L, Defilippo R, Bellusci S, Shi W, Driscoll B (2008). Stem/progenitor cells in lung development, injury repair, and regeneration. Proc Am Thorac Soc.

[27] Siegfried JM, Krishnamachary N, Gaither Davis A, Gubish C, Hunt JD, Shriver SP (1999). Evidence for autocrine actions of neuromedin B and gastrin-releasing peptide in non-small cell lung cancer. Pulm Pharmacol Ther.

