# Supplementary material for: Interaction between the estrogen receptor and fibroblast growth factor receptor pathways in non-small cell lung cancer
Source: Oncotarget. 2017 Mar 8;8(15):24063–76. doi: 10.18632/oncotarget.16030 (PMC5421827; doi:10.18632/oncotarget.16030)
Supplement: Supplementary file 1 [file oncotarget-08-24063-s001.pdf]

Interaction between the estrogen receptor and fibroblast growth factor receptor pathways in non-small cell lung cancer

Supplementary Material

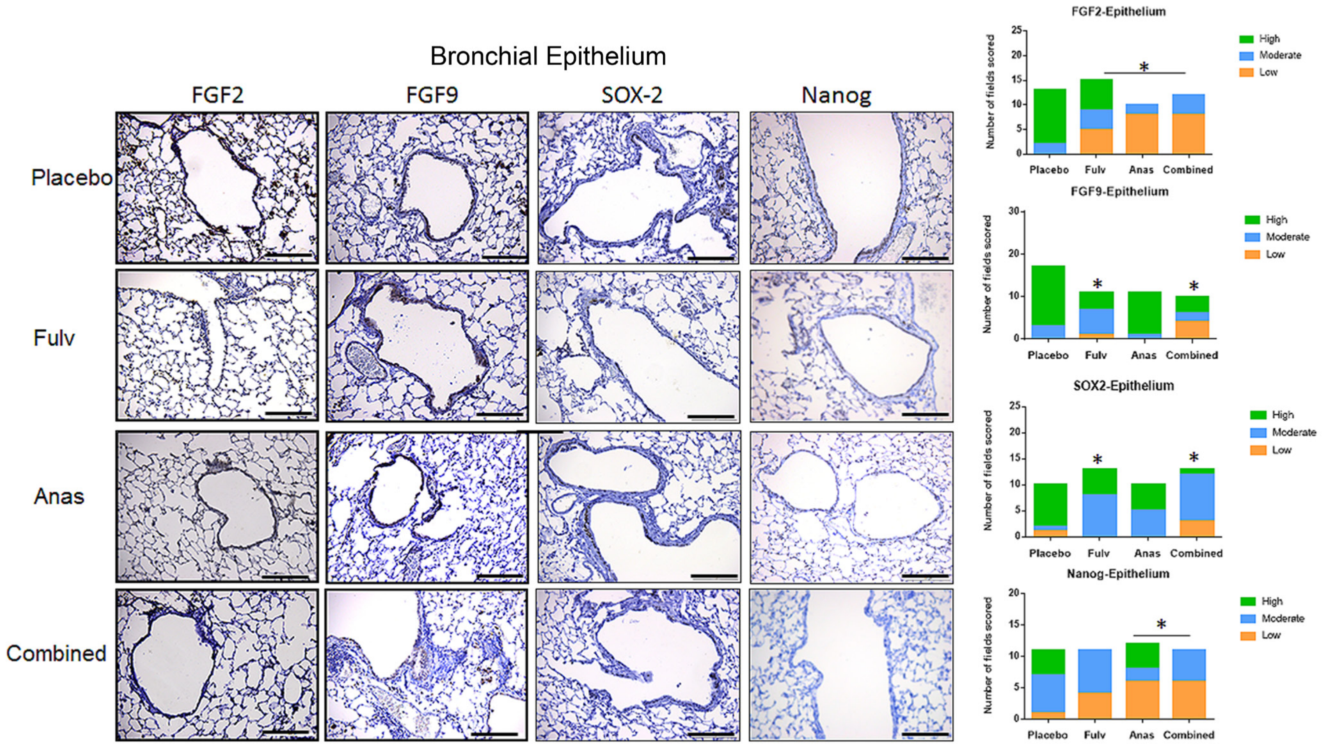

Supplementary Figure 1: Representative images and quantitation for FGFs and stem cell markers in the normal bronchial epithelium from lungs of mice exposed to NNK and treated with endocrine agents. FGF2 *P<0.02; FGF9, * P<0.018; Nanog, P<0.05; SOX2, * P<0.01. Scale bar= 143µm.

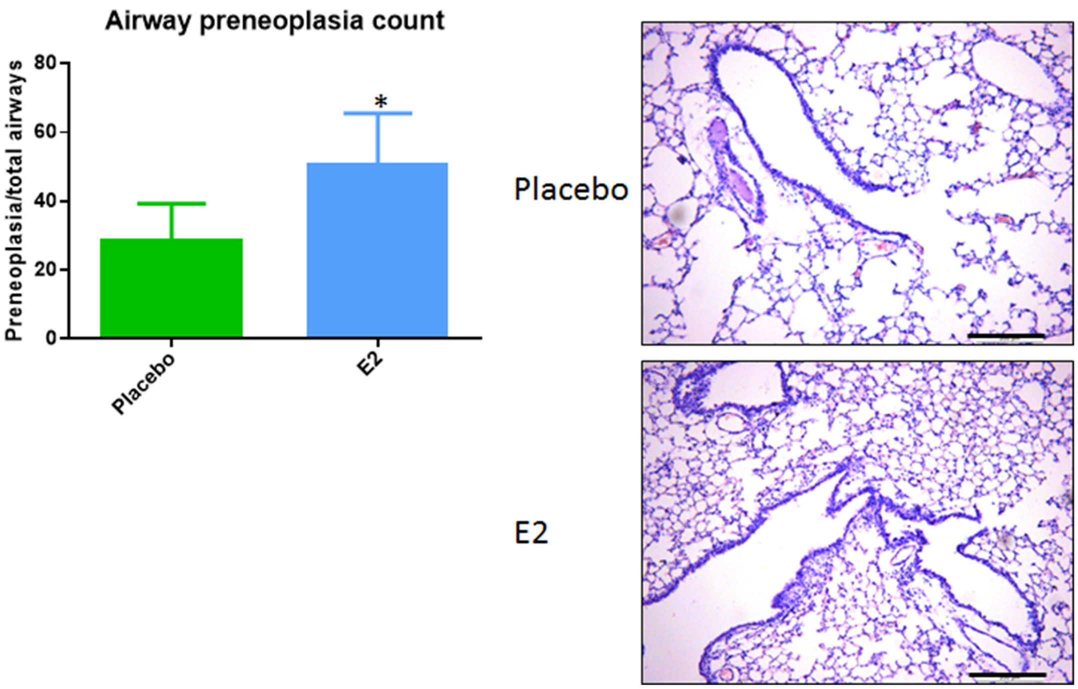

Supplementary Figure 2: Preneoplasia results in mice exposed to NNK with and without E2. Left, Airway preneoplasia counts. Right, Representative airways for placebo and E2 groups stained with hematoxylin and eosin. Scale bar= 200µm.

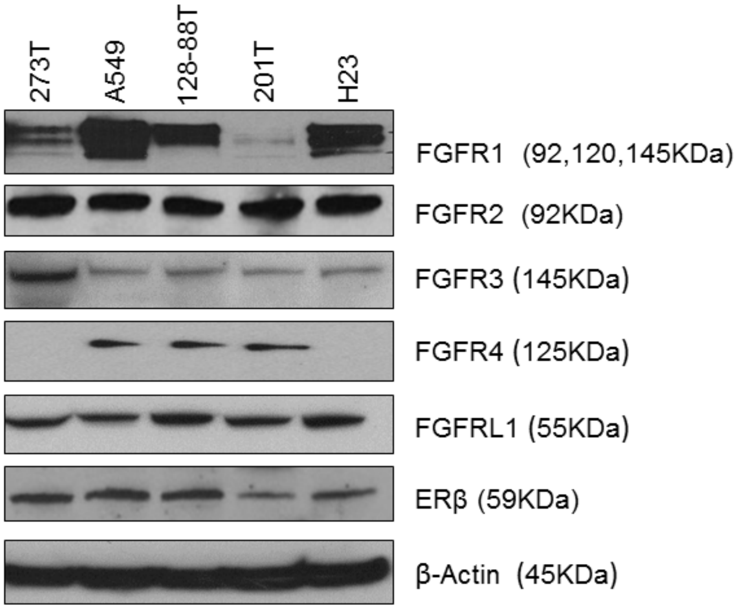

Supplementary Figure 3: FGFR and ERβ protein expression in NSCLC cell lines. FGFR1-5 and ERβ expression in NSCLC cell lines that lack FGFR genetic abnormalities. Representative immunoblot analysis is shown.

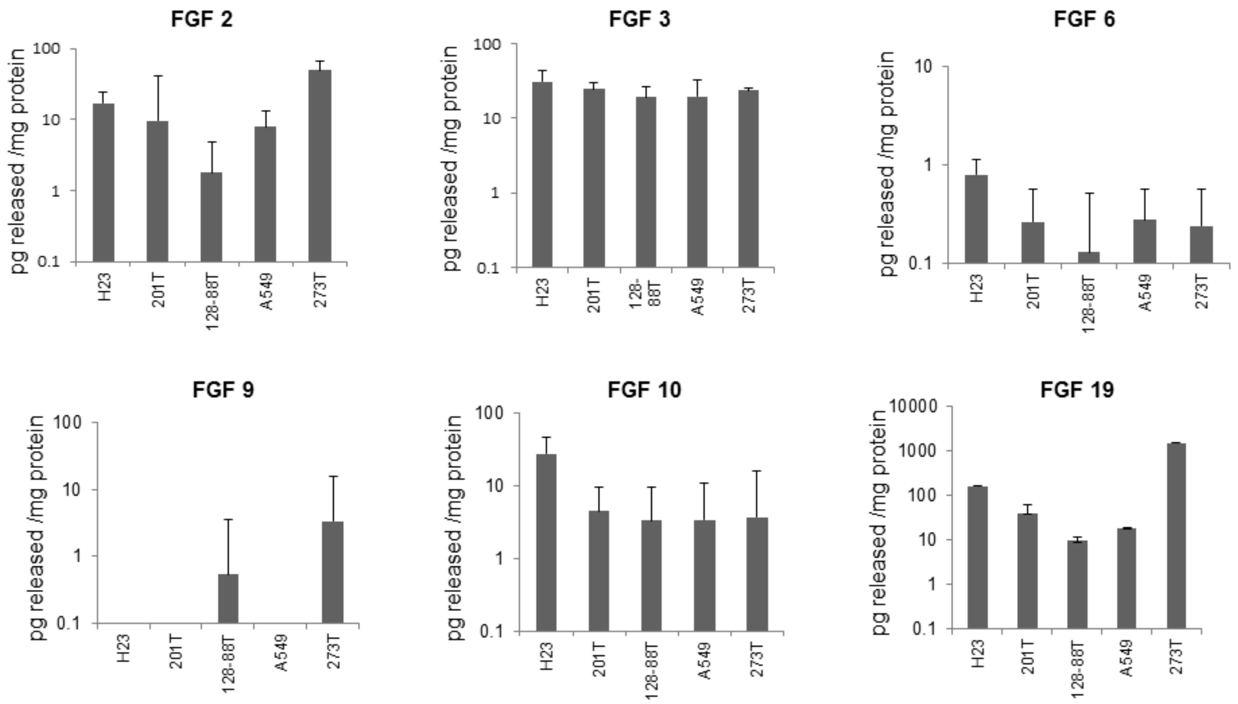

Supplementary Figure 4: FGF ligand release in NSCLC cell lines. ELISAs were performed for FGFs 2, 3, 4 6, 8, 9, 10 and 19 using concentrated media collected from each cell line. Level of FGF detected was normalized with the total protein content in the lysates of the cultured cells and results are presented as the mean pg released/mg protein ± S.E.

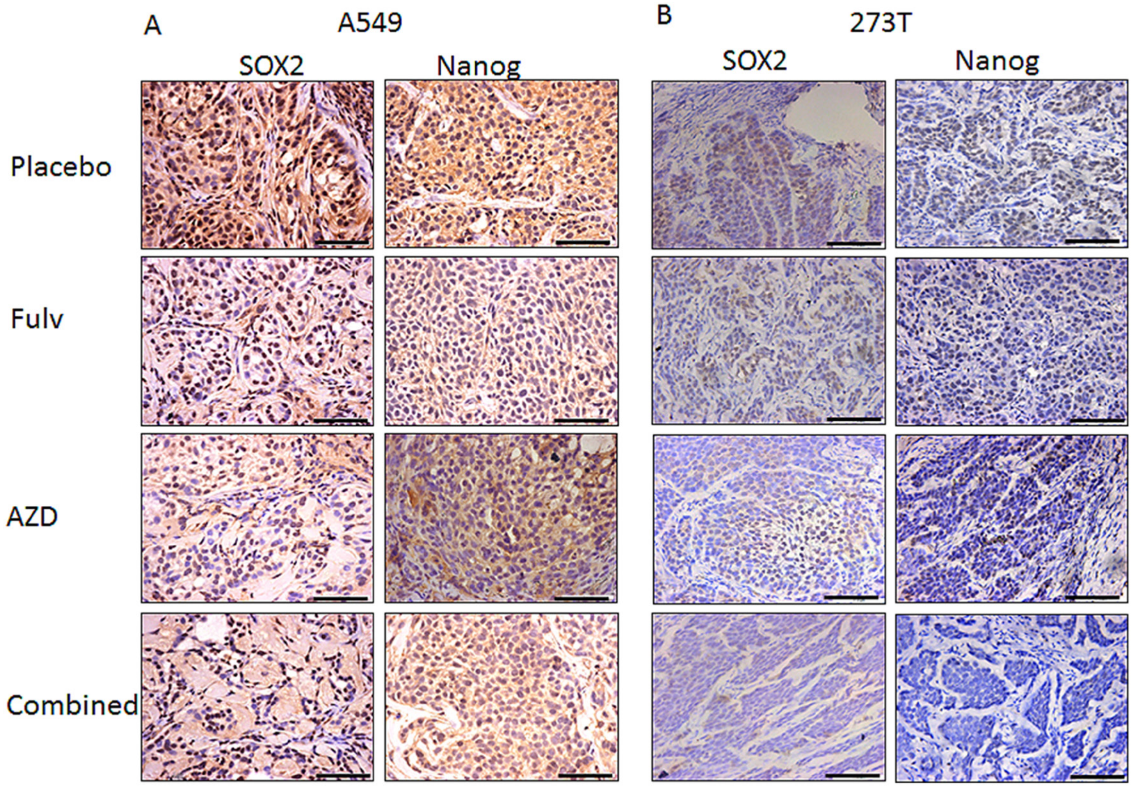

Supplementary Figure 5: Tumor sections from A549 and 273T xenografts stained for stem cell markers after treatment with fulvestrant and AZD4547. Representative images captured at 40X are shown. Scale bar= 71.4µm.

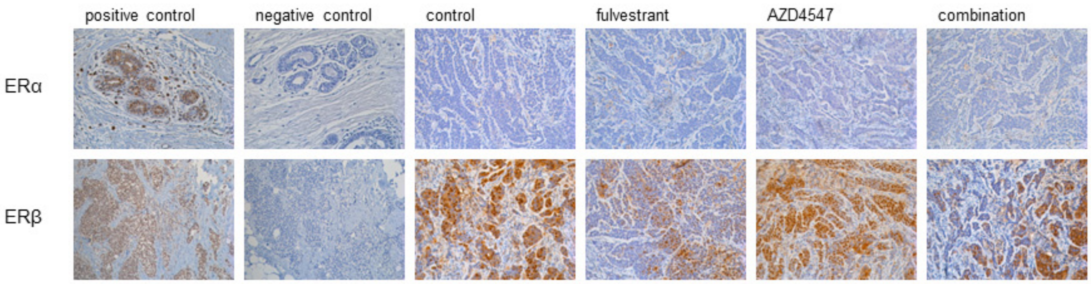

Supplementary Figure 6: ERα and ERβ expression in xenograft tumors. Representative IHC staining of ERα (antibody clone HC-20) and ERβ (antibody MCA1974ST) in 273T xenografts by treatment group. ER positive breast adenocarcinoma tissue was used as a positive control. Similar results were observed with A549 xenografts.

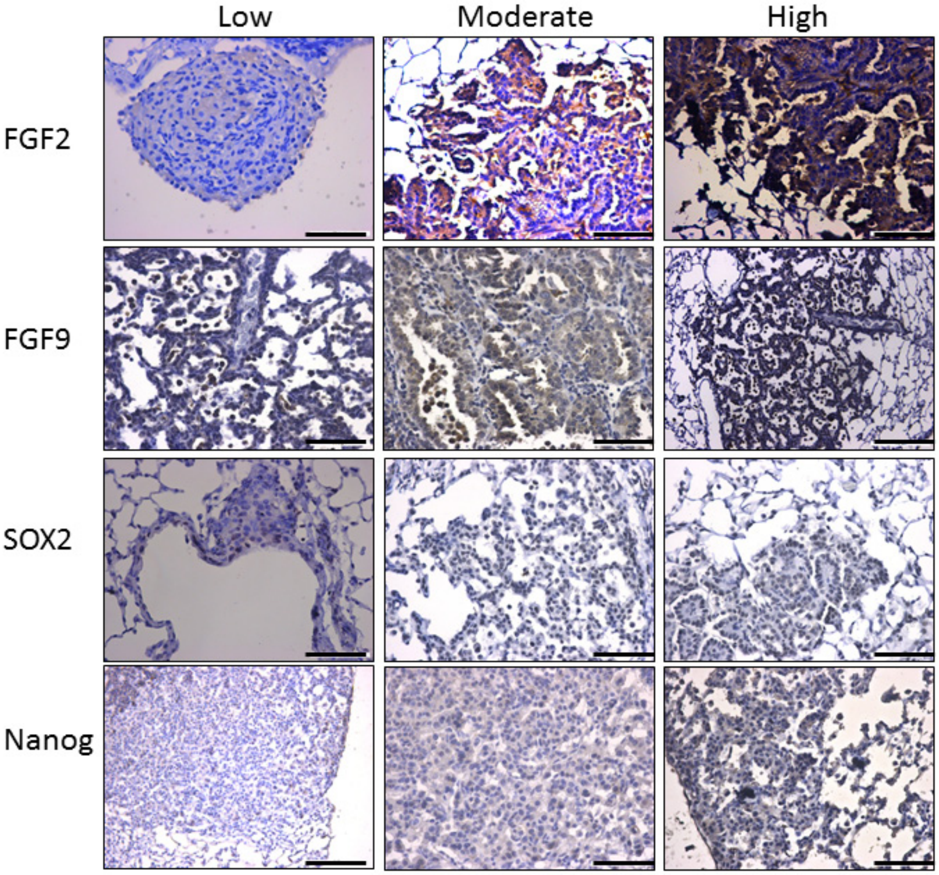

Supplementary Figure 7: Examples of low, moderate and high staining categories for each marker. Scale bar= 71.4μm.

Supplementary Table 1: Top ten differentially expressed genes in ERβhigh/ERβlow tumors

Symbol	Gene	P Value	Fold Change
ZNF334	Zinc finger protein 334	1.00E-06	2.7
FGFR5	FGF receptor-like 1 (decoy receptor)	8.00E-06	0.54
ARMCX1	Armadillo repeat-containing protein	1.40E-05	1.59
TNRSF1 3B	TNF receptor superfamily, member 13B	7.25E-03	0.35
POFUT1	Protein O-fucosyltransferase 1	2.77E-05	2.16
TNKS	Tankyrase	3.10E-04	1.84
SFTPB	Surfactant protein B	2.57E-03	0.38
FGFR1	FGF receptor 1	3.51E-03	2.41
PIAS	Protein inhibitor of STAT,1	5.90E-04	1.92
SLIT2	Slit homolog 2	3.59E-03	0.44
